# Evaluation of diversity and effective traits in Iranian cotton germplasm

**DOI:** 10.1371/journal.pone.0340581

**Published:** 2026-04-08

**Authors:** Hassan Najjar, Nafiseh Mahdi Nezhad, Majid Taherian, Barat Ali Fakheri, Maryam Allahdou, Mohammad Reza Ramazani Moghadam

**Affiliations:** 1 Department of Plant Breeding and Biotechnology, Faculty of Agriculture, University of Zabol, Zabol, Iran; 2 Horticulture Crop Research Department, Khorasan Razavi Agricultural and Natural Resources Research and Education Center, AREEO, Mashhad, Iran; KGUT: Graduate University of Advanced Technology, IRAN, ISLAMIC REPUBLIC OF

## Abstract

Drought is one of the most important abiotic factors influencing crop development and productivity. As a result, there is a need to enhance cultivars with high compatibility and low water requirements. This study examined the genetic variation of drought tolerance as well as the morphological, physiological, and agronomic aspects of cotton (*Gossypium hirsutum* L.) germplasm. For this reason, sixty-four genotypes of cotton were selected and cultivated in the field for two years under two moisture regimes, including normal and drought stress. All of the evaluated variables showed considerable genotypic variation between genotypes, indicating a great potential for trait improvement through focused selection in breeding programs. Drought stress has a detrimental influence on the majority of the assessed characteristics, reducing their genotypic variation. In both locations, most examined characteristics had quite high heritability estimates under both situations, indicating the presence of some important genes or QTLs influencing them. Principal component analysis revealed a negative association between earliness and photosynthetic and yield-related traits, indicating that selection for early maturity may reduce productivity. This suggests that selecting for earliness in cotton can decrease output. Contrasting genotypes identified by biplot analysis can be used to develop breeding populations for drought-tolerance studies.

## 1. Introduction

Cotton is one of the most valuable agricultural crops and a strategic fiber plant. It has significant economic importance and a strong commercial and agricultural position globally and in Iran, earning it the moniker “white gold” for its enormous economic significance [1,2]. Cotton, a glycophyte species, exhibits greater resilience to abiotic stressors than other major crops. However, cotton growth, yield, and fiber quality are affected by harsh environmental conditions such as drought. [3,4]. Producers should prioritize the identification of tolerant cultivars adapted to dry and semi-arid climates to minimize yield and quality loss [4,5].

Water resources in semi-arid and temperate regions are expected to become increasingly scarce due to climate change. Drought stress is a major environmental factor that impairs growth and development, reduces yield, and affects morphological, physiological, biochemical, and metabolic processes in plants [6–8]. Changing cultivation patterns and selecting drought-tolerant species with high adaptability and low water requirements are primary objectives of plant breeding in arid regions.

Due to changing climatic circumstances, drought stress has developed as a global concern that has a considerable impact on cotton output. According to estimates, drought stress has resulted in a loss of around 67% in cotton production, indicating that the detrimental effects of drought stress on cotton yield are more severe than other environmental pressures [9,10]. Improving water-use efficiency and implementing conservation strategies have become major concerns in irrigated agriculture. As a result, attempts have been made to better understand cotton’s adaptive mechanisms to drought stress [11,12]. Developing drought-tolerant cultivars remains a key objective of breeding programs and plays a critical role in climate-change mitigation [13].

Significant efforts have been undertaken to understand drought-tolerance mechanisms and identify drought-resistant traits in cotton [4]. A study of sixteen tetraploid cotton genotypes revealed substantial variation in yield and its components under drought conditions [14].

The three main requirements for producing stress-tolerant cultivars are accurate stress characterization, sufficient genetic variation for tolerance, and efficient screening techniques [15]. Information on genetic variability, heritability, and genotype–environment interactions assists breeders in improving genotypes [16,17]. Broad sense heritability estimations of variables can give information about the significance of genetic diversity accessible for selection by plant breeders in examined plant materials.

Yield in cotton is determined by multiple interacting factors influencing growth throughout the season, so direct selection is not always effective. [16]. In many plant species, models have been developed to predict yield and identify superior genotypes under normal and stress conditions [18,19]. Genetic information on yield and yield-related variables may be utilized to improve breeding program efficiency by finding appropriate indicators for choosing superior genotypes under both normal and stress situations [20]. Selecting appropriate traits with high genetic diversity is essential for effective indirect selection models. Hao et al. [19] developed an integrated criterion for drought resistance in maize that effectively identified drought-tolerant genotypes. The correlation analysis demonstrated that yield was positively associated with plant height, boll weight, number of open bolls, number of branches, and chlorophyll content under both conditions Saeidnia and Najjar reported a strong association between yield, plant height, number of bolls, and single boll weight in cotton, suggesting indirect selection can enhance yield [18]. Adopting cultivars that maintain yield and quality under drought is one of the most effective strategies to mitigate water stress in cotton. In other words, while developing stress-tolerant cotton cultivars, we should also consider yield stability. Enhancing and stabilizing cotton yield under normal and drought conditions is essential for global production and farmer adoption. To the best of our knowledge, limited information exists on genetic variation for drought-tolerance traits in cotton and the relationships among key attributes under normal and drought conditions The objectives of this study were to (i) evaluate cotton genotypes under drought stress and estimate genetic variation in agronomic, morphological, and physiological traits, (ii) analyze correlations among yield and related traits to propose an indirect selection index, and (iii) identify drought-tolerant genotypes in the studied germplasm. Overall, the studied germplasm exhibited wide genetic diversity, providing opportunities for breeding drought-tolerant cotton varieties.

## 2. Materials and methods

### 2.1. Experimental site

**Fig 1:**
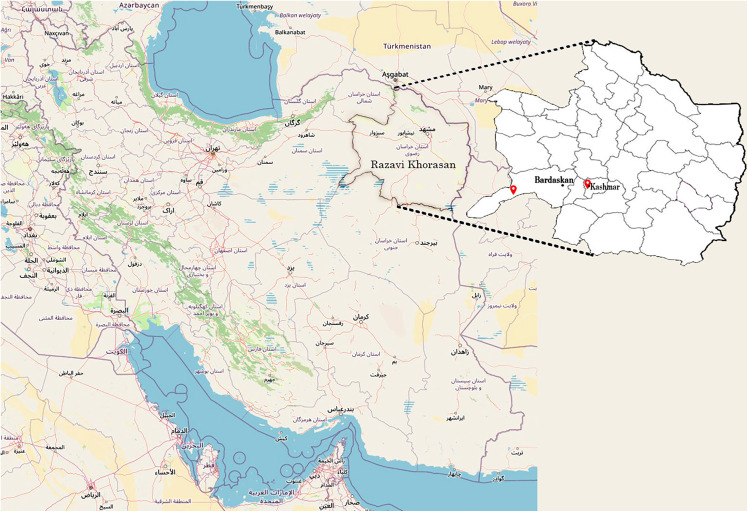
The map of Iran and the position of experimental sites of the study (https://www.usgs.gov/centers/eros/science/annual-national-land-cover-database).

This study was carried out during a two-year period (2021–2022) at the Agricultural and Natural Resources Research Station’s research farm in Kashmar, Iran (35° 11′ N, 58° 72′ E, 1063 m amsl) and a research farm in Bardaskan, Iran (34° 42′ N, 58° 15′ E, 985 m amsl) (Fig 1). The first location features silty loam soil with a pH of 7.8. The average annual precipitation and temperature in the region were 186 mm and 17.9°C, respectively (www.havairan.com). According to Koppen’s classification, this region is desert with a moderate climate. The second location contains loam soil with a pH of 8.0. The average annual precipitation and temperature in the region were 137 mm and 20.1°C, respectively (www.havairan.com). Koppen’s categorization indicates that this region has a hot and dry climate. Summers in this region are typically arid and rainless, necessitating irrigation.

### 2.2. Plant materials and field experiments

Sixty-four cotton genotypes (*Gossypium hirsutum*) derived from diallele crosses between eight different cotton cultivars were selected and cultivated in field trials for two years under two irrigation regimes: normal and drought stress. Hand-planted each genotype in plots of three 600 cm long rows, 70 cm row spacing, and 20 cm within-row spacing using a randomized complete block design with six replications. Three replications were assigned to each of the two moisture regimes (normal and drought stress). Under normal conditions, plants were watered after the root zone had consumed 50% of the total available soil water. Irrigation was carried out under the water stress situation when 90% of the total available soil water was drained from the root zone [21]. In each year of the experiment, the irrigation treatments were continuously applied during the growing season. Before applying drought stress, the reference evapotranspiration (ET_0_) was calculated using the FAO-Penman-Monteith method [21]. Amount of ET_0_ was determined by using daily meteorological data including: minimum and maximum temperature, wind speed, relative humidity and sunny hours that received from the Kashmar and Bardaskan Meteorological Station as part of the Iranian Meteorological Organization (http://www.weather.ir/). Crop evapotranspiration (ET_c_) was calculated using ET_0_ and modified crop coefficient (K_Mc_) provided by Allen et al. (1998) as follows:


$${\text{ET}}_{\text{C}}={\text{ET}}_{\text{0}}\times {\text{K}}_{\text{MC}}$$

where ET_0_ and ET_c_ are plant evapotranspiration under the field and container conditions (mm.day^-1^), respectively. K_MC_ is the microclimate coefficient and was assumed to be 1.2 [22]. When ΣETC reached to water allowable depletion depth (D_irrig_), it was the irrigation time for all irrigation levels. However, soil samples were taken every 2 days from different sites of each irrigation treatment at three depths (0–20, 20–40, and 40–60 cm) based on standard gravimetric methods [23] using a hand auger to determine the gravimetric soil-water content and detect the irrigation times. The water allowable depletion depth (D_irrig_) was calculated using the following formula:


$${\text{D}}_{\text{irrig}}=\left(\theta_{\text{FC}}-\theta_{\text{PWP}}\right)\times \text{MAD}\times \text{D}\times \left(\rho_{\text{b}}/\rho_{\text{w}}\right)$$

where θ_FC_ is soil gravimetric moisture percentage at field capacity (%), θ_PWP_ is soil gravimetric moisture percentage at wilting point (%), MAD is maximum allowable depletion (50% and 90% for normal irrigation and drought stress conditions, respectively), D is the root-zone depth (60 cm), ρ_b_ is the soil bulk density at root-zone (1.4 g.cm-3) and ρ_w_ is water density. Finally, the irrigation depth was determined according to the following equation:


$$I=\left[\theta_{FC}-\theta \right]\;\times D\times B$$

where I indicate irrigation depth (cm), θ_FC_ is soil gravimetric moisture percent at the field capacity, θ is soil gravimetric moisture percentage at the irrigating time, D is the root-zone depth (60 cm), B is and the soil bulk density at root-zone (1.4 g/cm3). The irrigation intervals during the growing season and between the two irrigation treatments were varied depending on the weather conditions. Water volume was calculated by multiplying the irrigation depth by surface area of plots. The drip-tape irrigation system was used to manage irrigation. The 16 mm drip-tape (Eurodrip S.A. Inc., Greece) with 20 centimeter dripper apart and 1.3 L h-1 flow rate for each dripper used for each crop row.

### 2.3. Measurements

In the present study, eighteen morphological, physiological, and agronomic parameters were examined under two levels of irrigation (normal and water stress) during 2021 and 2022. Leaf length (LL), leaf width (LW), crown diameter (CD), and plant height (PH) were measured on five plants from each genotype at maturity. Plant height (PH) was measured as the distance between the plant’s base and the top of its last bud at maturity. The plant base width was measured using a caliper based on crown diameter (CD). The number of sympodial branches (NSB), monopodial branches (NMB), closed bolls (NCB), and open bolls (NOB) were counted at maturity on five randomly selected plants from each genotype. A sensitive scale was used to measure the weight of each single boll. When more than 60% of the bolls in each plot bloomed, the cotton seed of each plant was harvested, and the weight of cotton seed yield per genotype was determined. The second harvest was completed around 20 days after the first harvest. The total yield was estimated by adding the yields of the first and second harvests. Earliness was determined as the ratio of the first harvest’s yield to the total output. Chlorophyll a (Chla) and chlorophyll b (Chlb) were measured with 80% acetone as a solvent [24] at wavelengths of 661.6 nm and 644.8 nm. The total chlorophyll content (TChl) was estimated as Chl a + Chlb. Then the chlorophyll a to chlorophyll b ratio (Chla/b; RAB) was computed. The technique described by Bates et al. [25] was used to measure proline from plant leaves. To measure chlorophyll and proline content, samples were taken from the leaves of the fifth sympodial branch.

### 2.4. Statistical analysis

The Kolmogorov-Smirnov test was used to assess data normality, while the Bartlett test was used to assess variance homogeneity. All data were analyzed using SAS software (version 9.4), and results were evaluated through analysis of variance and correlation tests. Data were treated as fixed for genotype effects and random for year effects. For significant F-values, trait means were compared using the least significant difference (LSD) test at *p* <.05 [26]. Variance components were computed using ANOVA mean squares that had been equated to their predicted variance components [27]. To determine association of traits, the phenotypic correlation coefficients were determined using SAS’s Proc CORR. Hallauer and Miranda [27] computed broad-sense heritability (h2b) (Eq. 1) using a mean phenotypic basis averaged over replications, years, and moisture environments:


$$\overset{\text{2}}{\underset{b}{h}}=\frac{\overset{\text{2}}{\underset{g}{\sigma }}}{\overset{\text{2}}{\underset{g}{\sigma }}+\frac{\overset{\text{2}}{\underset{ge}{\sigma }}}{e}+\frac{\overset{\text{2}}{\underset{gy}{\sigma }}}{y}+\frac{\overset{\text{2}}{\underset{gey}{\sigma }}}{ey}+\frac{\overset{\text{2}}{\underset{\delta }{\sigma }}}{re}+\frac{\overset{\text{2}}{\underset{\epsilon }{\sigma }}}{rey}}$$ (1)

where h^2^_b_ is the broad-sense heritability, $\overset{\text{2}}{\underset{g}{\sigma }}$ is the genotype, $\overset{\text{2}}{\underset{ge}{\sigma }}$ is the genotype × environment, $\overset{\text{2}}{\underset{gy}{\sigma }}$ is the genotype × year, $\overset{\text{2}}{\underset{gey}{\sigma }}$ is the genotype × environment × year variance; $\overset{\text{2}}{\underset{\delta }{\sigma }}$ and $\overset{\text{2}}{\underset{\epsilon }{\sigma }}$ are the error variance and the residual variance, respectively; while g, e, y, and r represent the number of genotypes, environments, years, and replications, respectively.

The data were also subjected to ANOVA for normal and water stress across years. Individual moisture environment (normal and water stress) data were used to evaluate variance components using SAS Proc MIXED. The broad-sense heritability on a mean phenotypic basis averaged over replications and years was determined using the following Eq. (2) [27]:


$$h^{\text{2}}=\frac{\overset{\text{2}}{\underset{g}{\sigma }}}{\overset{\text{2}}{\underset{g}{\sigma }}+\frac{\overset{\text{2}}{\underset{gy}{\sigma }}}{y}+\frac{\overset{\text{2}}{\underset{gr}{\sigma }}}{r}+\frac{\overset{\text{2}}{\underset{e}{\sigma }}}{ry}}$$ (2)

Where h^2^_b_ is the broad-sense heritability, $\overset{\text{2}}{\underset{g}{\sigma }}$ is the genotype, $\overset{\text{2}}{\underset{gy}{\sigma }}$ is the genotype × year, $\overset{\text{2}}{\underset{gr}{\sigma }}$ is the genotype × replication variance; and $\overset{\text{2}}{\underset{\text{e}}{\sigma }}$ and is the error variance; while g, y, and r indicate the number of genotypes, years, and replications, respectively. To determine the amount of genetic variation, the phenotypic coefficient of variation (PCV) (Eq. 3) and genotypic coefficient of variation (GCV) (Eq. 4) were estimated as:


$$\text{PCV}=\left(\sigma_{\text{p}}/\mu \right)\;\text{1}\text{0}\text{0}$$ (3)

$$\text{GCV}=\left(\sigma_{\text{g}}/\mu \right)\;\text{1}\text{0}\text{0}$$ (4)

where σ_p_ is the standard deviation of the phenotypic variance, σ_g_ is the standard deviation of the genotypic variance, and µ is the phenotypic mean [28]. Stepwise multiple linear regression was used according to Montgomery [29] to determine the variables accounting for the majority of total yield variation. Principal component analysis (PCA) was done based on a correlation matrix on all agro-morphological and physiological traits using Statgraphics software version 17.2 [30] and its biplots were drawn using R software version 4.3.

## 3. Results

The ANOVA results revealed significant variations in all of the evaluated qualities between the normal and drought stress settings in both sites, with the exception of earliness in Bardaskan (Tables 1 and 2). All measured variables exhibited significant genotypic variation, indicating substantial potential for trait improvement through selection in breeding programs. Tables 1 and 2 show substantial genotype × environment (GE) effects on all characteristics and locations. The ANOVA results showed that year and year × moisture environment had substantial influence on most attributes. Tables 1 and 2 show substantial genotype × year (G × Y) impacts on all examined characteristics.

**Table 1:** Combined ANOVA for measured traits in 64 genotypes of cotton evaluated at two environments (normal and drought stress) during 2 years in Kashmar region.

	df	BW	LP	YLD	ERL	LL	LW	LFS	CD	PH	NMB	NSB	NOB	NCB	CHLa	CHLb	TCHL	RAB	PRO
Year	1	74.45**	0.29ns	6344436.5**	3658.35**	64.79**	0.29ns	0.53*	0.0002ns	2.88ns	0.05ns	134.18**	24.10**	12.76**	0.0001ns	0.00005ns	0.0000001ns	0.004ns	2.78**
Environment	1	395.05**	603.85**	298739190**	187.87**	499.7**	764.06**	69.9**	7.16**	20781.28**	5.07**	1493.35**	1296.8**	0.74**	6.42**	0.56**	12.5**	7.18**	6730.98**
Year*Env	1	8.65**	65.92**	8846797.6**	4842.84**	136.05**	466.23**	430.61**	0.0002ns	4.85ns	2.97*	495.8**	61.91**	61.34**	0.008**	0.00001ns	0.008*	0.14*	0.45*
Block	8	0.03ns	2.13ns	140408.3*	4.19ns	0.35ns	0.32ns	0.05ns	0.01ns	63.45ns	0.17ns	0.25ns	0.1ns	0.01ns	0.0003ns	0.00003ns	0.0005ns	0.006ns	0.05ns
Treat	63	1.74**	25.16**	1497194.1**	93.37**	4.17**	3.70**	2.13**	0.04**	188.54**	0.99**	8.63**	11.63**	1.8**	0.067**	0.005**	0.11**	0.4**	16.02**
Env*Treat	63	0.59**	28.66**	432182.1**	106.98**	2.95**	3.42**	1.23**	0.03**	134.94**	1.45**	3.67**	9.2**	1.69**	0.017**	0.002**	0.03**	0.18**	8.13**
Year*Treat	63	1.1**	12.91**	398982**	118.11**	3.77**	3.50**	1.42**	0.02*	109.93**	1.24**	3.68**	9.8**	1.74**	0.003**	0.0006**	0.005**	0.1**	0.82**
Year*Env*Treat	63	0.63**	23.06**	342118.7**	65.97**	3.33**	1.74**	1.7**	0.03**	132.49**	1.04**	3.85**	9.23**	2.17**	0.0018**	0.0004**	0.002**	0.1**	0.76**
Error	504	0.04	1.56	41572	14.23	0.28	0.45	0.06	0.01	38.86	0.34	0.69	0.74	0.009	0.0005	0.0001	0.0006	0.023	0.12

* and **: Significant at the 0.05 and 0.01 probability level, respectively; ns: not significant.

BW: boll weight; CD: crown diameter; CHLa: chlorophyll a; CHLb: chlorophyll b; ERL: earliness; LFS: LL: leaf length; LP: lint percentage; LW: leaf width; NCB: number of closed bolls; NMB: number of monopodial branches; NOB: number of opened bolls; NSB: number of sympodial branches; PH: plant height; PRO: proline content; RAB: ratio of chlorophyll a to chlorophyll b; TCHL: total chlorophyll.

**Table 2:** Combined ANOVA for measured traits in 64 genotypes of cotton evaluated at two environments (normal and drought stress) during 2 years in Bardaskan region.

	df	BW	LP	YLD	ERL	LL	LW	LFS	CD	PH	NMB	NSB	NOB	NCB	CHLa	CHLb	TCHL	RAB	PRO
Year	1	2.04**	350.73**	926203.96**	249.11**	2.39*	0.6ns	1.08**	0.00007ns	0.0058ns	0.07ns	110.15**	0.047ns	0.16**	0.00003ns	0.0012**	0.0014*	0.25**	0.23*
Environment	1	377.09**	84.67**	87483098.28**	20.88ns	201.29**	685.2**	65.58**	7.48**	18114.98**	7.27**	899.43**	1575.38**	5.03**	3.17**	1.14**	9.35**	15.69**	3366.54**
Year*Env	1	8.84**	40.8**	249865.19*	916.39**	0.05ns	2.93*	0.09ns	0.003ns	0.88ns	0.037ns	129.15**	1.02ns	0.1**	0.0007*	0.00006ns	0.001ns	0.046ns	0.21*
Block	8	0.004ns	0.36ns	18500.67ns	19.1ns	0.11ns	0.18ns	0.034ns	0.003ns	14.2ns	0.067ns	0.35ns	0.54ns	0.002ns	0.00009ns	0.00003ns	0.00006ns	0.017ns	0.015*
Treat	63	1.96**	15.32**	872424.72**	334.1**	2.65**	1.9**	1.14**	0.013**	66.19**	0.29**	5.22**	8.05**	1.3**	0.024**	0.002**	0.04**	0.3**	6.52**
Env*Treat	63	0.49**	23.67**	176956.61**	162.95**	1.25**	2.45**	1.9**	0.01*	96.87**	0.39**	3.1**	7.6**	0.79**	0.007**	0.001**	0.012**	0.17**	2.9**
Year*Treat	63	0.66**	18.59**	438672.39**	174.82**	0.99**	1.39**	1.92**	0.011*	73.42**	0.28**	2.6**	6.83**	0.85**	0.0011**	0.0003**	0.0016**	0.09**	0.28**
Year*Env*Treat	63	0.51**	22.36**	197148.14**	226.6**	0.98**	2.63**	1.01**	0.015**	106.29**	0.31**	4.22**	6.44**	1.34**	0.001**	0.0003**	0.0015**	0.09**	0.32**
Error	504	0.028	1.35	20481.6	15.26	0.175	0.318	0.05	0.007	21	0.079	0.59	0.72	0.006	0.00027	0.00006	0.0004	0.018	0.05

* and **: Significant at the 0.05 and 0.01 probability level, respectively; ns: not significant.

BW: boll weight; CD: crown diameter; CHLa: chlorophyll a; CHLb: chlorophyll b; ERL: earliness; LFS: LL: leaf length; LP: lint percentage; LW: leaf width; NCB: number of closed bolls; NMB: number of monopodial branches; NOB: number of opened bolls; NSB: number of sympodial branches; PH: plant height; PRO: proline content; RAB: ratio of chlorophyll a to chlorophyll b; TCHL: total chlorophyll.

In both locations, the data were analyzed for normal and drought-stress conditions across years. Drought stress adversely affected most measured traits except for proline (PRO), reducing genotypic variation. (Tables 3 and 4). Under drought stress, yields declined by 27.45% and 22.54% in Kashmar and Bardaskan, respectively. Earliness decreased slightly under drought stress, whereas proline content increased markedly. Under water stress conditions, proline (PRO) levels increased by 185.31% and 212.18% in Kashmar and Bardaskan, respectively (Tables 3 and 4).

**Table 3:** Phenotypic coefficient of variation (PCV), genotypic coefficient of variation (GCV), broad-sense heritability, and the effect of drought stress on different traits of cotton during 2 years in Kashmar.

Traits	Mean ± SE		Decrease (%)	PCV(%)		GCV(%)		h^2^_b_(%)	
	Normal	Stress		Normal	Stress	Normal	Stress	Normal	Stress
BW	5.42	3.99	26.38**	24.70	27.01	22.73	22.60	84.71	70.01
LP	32.59	30.82	5.43**	18.52	18.19	16.78	15.85	82.18	75.92
YLD	4544.91	3297.54	27.45**	26.08	27.56	24.77	24.66	90.18	80.10
ERL	63.86	62.87	1.55**	16.64	20.59	14.62	16.91	77.22	67.45
LL	8.97	7.36	17.95**	24.23	30.81	20.29	26.53	70.18	74.15
LW	10.57	8.58	18.83**	19.77	26.31	17.44	22.48	77.81	72.97
LFS	4.49	3.89	13.36**	34.64	36.96	29.51	32.57	72.58	77.65
CD	1.54	1.34	12.99**	13.93	18.35	10.58	16.13	57.74	77.28
PH	86.93	76.53	11.96**	15.84	21.32	12.60	18.63	63.25	76.39
NMB	2.09	1.92	8.13**	40.83	79.42	12.27	69.44	59.04	76.45
NSB	13.71	10.92	20.35**	21.82	23.85	19.36	20.98	78.75	77.43
NOB	14.85	12.25	17.51**	25.94	29.57	22.13	25.59	72.83	74.88
NCB	1.74	1.80	−3.45**	86.94	88.03	74.89	74.35	74.19	71.34
CHL a	0.65	0.46	29.23**	34.61	41.38	34.30	40.55	98.19	96.02
CHL b	0.25	0.19	24.00**	18.29	40.98	17.46	40.06	91.10	95.54
TCHL	0.94	0.68	27.66**	29.22	39.74	28.98	39.14	98.42	97.01
RAB	2.60	2.41	7.31**	22.96	23.55	21.77	21.19	89.9	81.01
PRO	3.20	9.13	−185.31**	60.85	50.27	60.36	49.51	98.40	96.99

BW: boll weight; CD: crown diameter; CHLa: chlorophyll a; CHLb: chlorophyll b; ERL: earliness; LFS: LL: leaf length; LP: lint percentage; LW: leaf width; NCB: number of closed bolls; NMB: number of monopodial branches; NOB: number of opened bolls; NSB: number of sympodial branches; PH: plant height; PRO: proline content; RAB: ratio of chlorophyll a to chlorophyll b; TCHL: total chlorophyll.

**Table 4:** Phenotypic coefficient of variation (PCV), genotypic coefficient of variation (GCV), broad-sense heritability, and the effect of drought stress on different traits of cotton during 2 years in Bardaskan.

Traits	Mean ± SE		Decrease (%)	PCV(%)		GCV(%)		h^2^_b_(%)	
	Normal	Stress		Normal	Stress	Normal	Stress	Normal	Stress
BW	4.79	3.39	29.23**	27.99	30.37	26.15	27.52	87.27	82.13
LP	31.58	30.91	2.12**	15.59	17.76	13.34	14.89	73.25	70.31
YLD	2995.22	2320.21	22.54**	28.74	31.88	25.47	29.46	78.52	85.39
ERL	64.09	63.76	0.51 ns	27.68	28.76	23.04	26.19	69.28	82.93
LL	7.29	6.27	13.99**	24.34	18.25	20.25	15.03	69.28	67.80
LW	9.64	7.75	19.61**	18.12	22.59	15.54	18.73	73.49	68.77
LFS	3.64	3.06	15.93**	42.00	43.07	38.70	33.48	84.90	60.43
CD	1.27	1.08	14.96**	11.29	13.39	89.50	96.59	62.80	52.01
PH	65.90	56.18	14.75**	18.18	18.43	14.66	14.85	65.00	64.93
NMB	1.33	1.14	14.29**	44.20	70.27	31.88	62.56	52.02	79.25
NSB	12.43	10.27	17.38**	19.08	23.34	16.26	20.04	72.65	73.68
NOB	14.10	11.24	20.28**	23.21	28.53	19.81	24.88	72.85	76.04
NCB	1.66	1.50	9.64**	72.52	78.65	63.31	66.59	76.21	71.69
CHL a	0.47	0.34	27.66**	27.19	36.29	26.77	35.67	96.91	96.60
CHL b	0.22	0.14	36.36**	15.40	32.37	14.72	31.01	91.31	91.79
TCHL	0.73	0.50	31.51**	22.52	33.08	22.24	32.56	97.49	96.86
RAB	2.17	2.45	−12.90**	19.22	25.22	18.04	23.07	88.13	83.71
PRO	1.97	6.15	−212.18**	62.65	46.47	60.55	45.98	93.42	97.93

BW: boll weight; CD: crown diameter; CHLa: chlorophyll a; CHLb: chlorophyll b; ERL: earliness; LFS: length of fifth sympodial; LL: leaf length; LP: lint percentage; LW: leaf width; NCB: number of closed bolls; NMB: number of monopodial branches; NOB: number of opened bolls; NSB: number of sympodial branches; PH: plant height; PRO: proline content; RAB: ratio of chlorophyll a to chlorophyll b; TCHL: total chlorophyll.

Phenotypic and genotypic coefficients of variation (PCV and GCV) for normal and drought stress environments are given in Tables 3 and 4. According to GCV, NMB had a wider range of genetic variation in Kashmar, whereas YLD and BW had a lesser range of genetic variation (Table 3). In Bardaskan, the NMB range was higher, while the PH and CD ranges were lower. Broad-sense heritability estimates for each moisture environment are presented in Tables 3 and 4. According to the findings, some assessed characteristics had greater heritability estimates in normal conditions than in drought stress conditions, whereas the other traits had higher heritability estimates under drought stress (Tables 3 and 4).

Phenotypic correlation coefficients, calculated from the averages of data collected over two years in normal and drought stress conditions for Kashmar and Bardaskan regions, are presented in Tables 5 and 6, respectively. Under normal conditions, yield in the Kashmar region correlated significantly and positively with BW, ERL, CHL a, CHL b, TCHL, ratio of CHLa to CHLb (RAB), and PRO, but negatively with NCB. Under drought stress, it was favorably connected with BW, LFS, PH, NSB, NOB, NCB, CHL a, CHL b, TCHL, and PRO, but negatively correlated with ERL and LL. Under normal conditions, earliness had significant and positive correlations with LP, YLD, and RAB, but a negative correlation with NCB; under drought stress conditions, it was positively correlated with PRO, and negatively associated with BW, YLD, LFS, PH, NSB, NOB, NCB, CHL a, CHL b, and TCHL (Table 5). Under normal conditions, yield in the Bardaskan region correlated significantly and positively with BW, ERL, CHL a, CHL b, TCHL, RAB, and PRO, but negatively with LL. Under drought stress, it was favorably connected with BW, CHL a, CHL b, TCHL, and PRO, but negatively correlated with ERL and PH. Under normal conditions, earliness had significant and positive correlations with BW, YLD, CHL a, CHL b, and TCHL, and a negative correlation with LL; however, under drought stress conditions, it was positively correlated with PH and PRO, and negatively associated with YLD, NOB, CHL a, CHL b, and TCHL (Table 6).

**Table 5:** Correlation coefficients between different traits under non- stress (above diagonal) and drought stress (below diagonal) conditions over 2 years in Kashmar.

	BW	LP	YLD	ERL	LL	LW	LFS	CD	PH	NMB	NSB	NOB	NCB	CHLa	CHLb	TCHL	RAB	PRO
BW	1	0.222	0.363**	0.066	−0.007	−0.063	−0.110	−0.049	−0.052	0.332**	0.217	0.036	−0.085	0.581**	0.581**	0.594**	0.456**	0.722**
LP	0.205	1	0.098	0.346**	−0.100	−0.189	−0.052	−0.028	−0.093	−0.138	0.139	0.062	−0.176	0.217	0.094	0.201	0.277*	0.209
YLD	0.567**	0.102	1	0.293*	−0.151	−0.104	0.002	0.044	−0.163	0.184	0.085	0.146	−0.274*	0.731**	0.615**	0.728**	0.673**	0.603**
ERL	−0.390**	−0.231	−0.737**	1	−0.208	−0.233	0.109	−0.236	−0.130	0.041	−0.061	0.042	−0.415**	0.243	0.146	0.233	0.292*	0.154
LL	−0.036	−0.025	−0.281*	0.206	1	0.088	0.138	0.079	0.317*	−0.153	0.076	−0.055	0.173	0.072	−0.055	0.052	0.140	0.023
LW	−0.090	−0.070	−0.223	0.187	0.290*	1	0.283*	0.008	0.043	0.007	−0.108	0.062	0.171	−0.144	0.011	−0.122	−0.254*	−0.127
LFS	0.275*	0.309*	0.371**	−0.360**	−0.179	0.077	1	0.082	−0.017	0.149	0.134	−0.032	0.028	0.110	0.132	0.117	0.069	−0.064
CD	0.095	−0.011	−0.158	0.208	0.153	−0.048	−0.044	1	0.038	0.183	0.007	0.169	0.141	0.038	0.024	0.036	0.034	0.099
PH	0.210	0.074	0.466**	−.479**	−0.140	−0.031	0.303*	−0.110	1	0.107	0.076	−0.138	0.054	−0.047	−0.148	−0.065	0.020	−0.086
NMB	0.034	−0.209	0.004	0.006	−0.054	−0.275*	−0.094	0.030	−0.077	1	0.100	0.002	−0.076	0.248*	0.248*	0.253*	0.191	0.314*
NSB	0.381**	0.162	0.650**	−0.713**	−0.236	−0.095	0.425**	−0.135	0.487**	0.010	1	0.138	−0.098	0.474**	0.414**	0.474**	0.413**	0.071
NOB	0.337**	0.172	0.672**	−0.676**	−0.224	−0.188	0.357**	−0.135	0.395**	0.005	0.734**	1	0.048	0.109	0.146	0.118	0.060	0.078
NCB	0.108	0.108	0.357**	−0.495**	−0.047	−0.090	0.159	−0.144	0.337**	0.112	0.487**	0.512**	1	−0.166	−0.126	−0.164	−0.172	0.000
CHLa	0.595**	−0.006	0.651**	−0.491**	−0.137	−0.139	0.385**	−0.009	0.428**	−0.024	0.734**	0.545**	0.275*	1	0.845**	0.996**	0.905**	0.751**
CHLb	0.484**	−0.100	0.549**	−0.392**	−0.088	−0.161	0.204	−0.050	0.346**	0.032	0.543**	0.430**	0.204	0.851**	1	0.890**	0.541**	0.686**
TCHL	0.580**	−0.035	0.641**	−0.477**	−0.127	−0.150	0.343**	−0.022	0.417**	−0.008	0.700**	0.528**	0.263*	0.987**	0.924**	1	0.864**	0.757**
RAB	0.228	0.179	0.197	−0.194	−0.096	0.043	0.345**	0.062	0.151	−0.106	0.370**	0.227	0.147	0.310*	−0.233	0.156	1	0.635**
PRO	0.606**	−0.025	0.652**	−0.474**	−0.153	−0.191	0.311*	−0.025	0.414**	0.009	0.691**	0.548**	0.252*	0.949**	0.901**	0.965**	0.128	1

BW: boll weight; CD: crown diameter; CHLa: chlorophyll a; CHLb: chlorophyll b; ERL: earliness; LFS: LL: length of fifth sympodial; leaf length; LP: lint percentage; LW: leaf width; NCB: number of closed bolls; NMB: number of monopodial branches; NOB: number of opened bolls; NSB: number of sympodial branches; PH: plant height; PRO: proline content; RAB: ratio of chlorophyll a to chlorophyll b; TCHL: total chlorophyll.

**Table 6:** Correlation coefficients between different traits under non- stress (above diagonal) and drought stress (below diagonal) conditions over 2 years in Bardaskan.

	BW	LP	YLD	ERL	LL	LW	LFS	CD	PH	NMB	NSB	NOB	NCB	CHLa	CHLb	TCHL	RAB	PRO
BW	1	0.009	0.452**	0.355**	−.254*	0.017	−0.038	−0.006	0.032	0.034	0.329**	0.070	0.097	0.522**	0.375**	0.512**	0.462**	0.458**
LP	0.062	1	0.096	0.106	0.008	0.003	0.114	0.054	−0.135	−0.043	0.033	0.042	−0.033	0.022	−0.018	0.014	0.038	0.086
YLD	0.427**	0.117	1	0.450**	−0.478**	−0.219	−0.089	−0.084	0.055	0.111	0.174	−0.035	−0.003	0.637**	0.531**	0.640**	0.520**	0.641**
ERL	−0.075	−0.062	−0.449**	1	−0.344**	−0.221	−0.109	0.031	0.142	0.176	−0.006	−0.055	0.095	0.274*	0.316*	0.294*	0.173	0.210
LL	0.048	−0.078	−0.206	−0.045	1	0.465**	0.161	−0.067	−0.128	−0.145	−0.109	0.029	0.077	−0.275*	−0.213	−0.273*	−0.264*	−0.358**
LW	−0.097	−0.030	0.100	−0.159	−0.018	1	0.117	−0.006	−0.171	−0.016	0.021	0.009	−0.089	−0.113	0.015	−0.091	−0.211	−0.073
LFS	0.265*	−0.028	0.133	−0.021	0.085	−0.034	1	0.054	−0.169	−0.082	−0.139	−0.128	0.100	−0.233	−0.065	−0.207	−0.297*	−0.199
CD	−0.004	0.049	−0.047	−0.114	−0.031	−0.062	−0.277*	1	−0.137	−0.185	−0.034	−0.044	0.065	−0.013	−0.008	−0.012	−0.023	0.022
PH	−0.347**	−0.110	−0.530**	0.379**	0.154	0.120	−0.105	0.042	1	0.069	−0.022	−0.074	0.066	−0.020	−0.091	−0.036	0.059	−0.050
NMB	0.089	0.064	0.115	0.063	−0.075	−0.117	0.022	0.100	0.034	1	−0.142	0.038	−0.060	0.030	0.032	0.032	0.034	0.081
NSB	0.486**	0.152	0.205	−0.142	0.209	−0.149	0.106	−0.104	−0.236	−0.109	1	0.141	−0.121	0.500**	0.432**	0.505**	0.359**	0.374**
NOB	0.298*	0.018	0.240	−0.302*	0.001	0.017	0.369**	−0.121	−0.032	0.082	0.116	1	−0.100	−0.029	−0.021	−0.029	−0.021	0.084
NCB	−0.024	−0.087	−0.112	0.085	0.058	−0.071	0.069	−0.100	0.212	−0.094	−0.020	0.146	1	−0.105	−0.045	−0.096	−0.117	−0.033
CHLa	0.709**	0.204	0.649**	−0.278*	−0.015	−0.045	0.234	−0.044	−0.431**	0.116	0.528**	0.218	−0.029	1	0.766**	0.991**	0.842**	0.826**
CHLb	0.455**	0.137	0.640**	−0.190	−0.105	−0.035	0.014	0.050	−0.309*	0.108	0.343**	0.131	−0.035	0.743**	1	0.846**	0.302*	0.703**
TCHL	0.677**	0.196	0.681**	−0.268*	−0.041	−0.044	0.186	−0.021	−0.421**	0.120	0.505**	0.206	−0.032	0.983**	0.854**	1	0.763**	0.833**
RAB	0.459**	0.130	0.134	−0.158	0.113	−0.034	0.301*	−0.116	−0.231	0.029	0.342**	0.134	0.013	0.530**	−0.169	0.365**	1	0.640**
PRO	0.685**	0.256*	0.677**	−0.272*	−0.076	−0.049	0.155	0.062	−0.474**	0.065	0.510**	0.131	−0.065	0.947**	0.774**	0.950**	0.418**	1

BW: boll weight; CD: crown diameter; CHLa: chlorophyll a; CHLb: chlorophyll b; ERL: earliness; LFS: length of fifth sympodial; LL: leaf length; LP: lint percentage; LW: leaf width; NCB: number of closed bolls; NMB: number of monopodial branches; NOB: number of opened bolls; NSB: number of sympodial branches; PH: plant height; PRO: proline content; RAB: ratio of chlorophyll a to chlorophyll b; TCHL: total chlorophyll.

The stepwise multiple linear regression method was used to identify the factors that account for the majority of yield variation. In the Kashmar region and under non-stress conditions, boll weight and number of closed bolls explained 44% of the overall variance in yield (Table 7), with boll weight being the most relevant component (Partial R2 = 36%). In the stress condition, boll weight and leaf length explained 48% of the observed variance in yield (Table 7). The other variables were excluded from the analysis due to their low relative contributions. Under drought stress, boll weight was the most important factor in yield variation (Partial R2 = 36%). In Bardaskan and under normal conditions, leaf length and boll weight explained 35% of the total variation in yield (Table 8), with boll weight being the most important factor (Partial R2 = 23%). In the stress condition, boll weight and leaf length explained 23% of the observed variation in yield (Table 8). Under these conditions, leaf length was the most important factor influencing yield.

**Table 7:** Results from stepwise regression analysis for predicting yield of cotton genotypes evaluated under normal and drought stress conditions in Kashmar.

Treatment	Variable entered	Parameter estimate	Partial R^2^	Model R^2^	F value
Normal	Boll weight	9.85	0.36	0.36	9.42**
	Number of closed bolls	4.88	0.08	0.44	7.21**
	Intercept	45.57	–	–	0.31 n.s
Stress	Boll weight	5.59	0.36	0.36	9.14**
	Leaf length	−10.46	0.12	0.48	9.31**
	Intercept	5.29	–	–	0.04 n.s

* and ** show significance at the 0.05 and 0.01 probability levels, respectively.

**Table 8:** Results from stepwise regression analysis for predicting yield of cotton genotypes evaluated under normal and drought stress conditions in Bardaskan.

Treatment	Variable entered	Parameter estimate	Partial R^2^	Model R^2^	F value
Normal	Leaf length	9.85	0.23	0.23	18.39**
	Boll weight	4.88	0.12	0.35	16.09**
	Intercept	45.57	–	–	0.31 n.s
Stress	Boll weight	5.59	0.36	0.18	13.86**
	Leaf length	−10.46	0.12	0.23	9.34**
	Intercept	5.29	–	–	0.04 n.s

* and ** show significance at the 0.05 and 0.01 probability levels, respectively.

**Fig 2:**
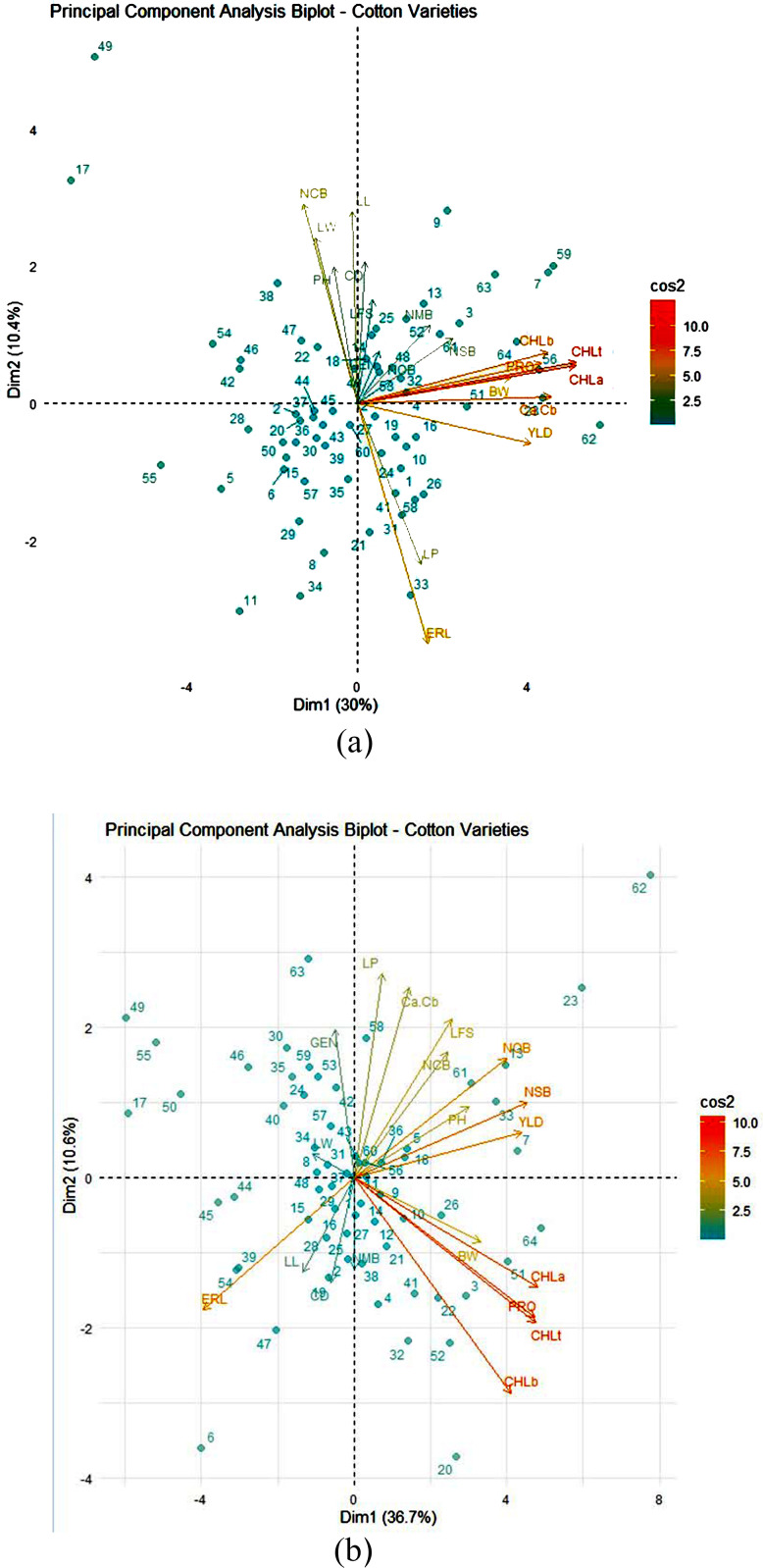
Distribution of the first two principal components (PC) of morphological, agronomic and physiological traits averaged over two years in 64 genotypes of cotton under (a) normal and (b) drought stress conditions in Kashmar region. BW: boll weight; CD: crown diameter; CHLa: chlorophyll a; CHLb: chlorophyll b; ERL: earliness; LFS: length of fifth sympodial; LL: leaf length; LP: lint percentage; LW: leaf width; NCB: number of closed bolls; NMB: number of monopodial branches; NOB: number of opened bolls; NSB: number of sympodial branches; PH: plant height; PRO: proline content; RAB: ratio of chlorophyll a to chlorophyll b; TCHL: total chlorophyll.

**Fig 3:**
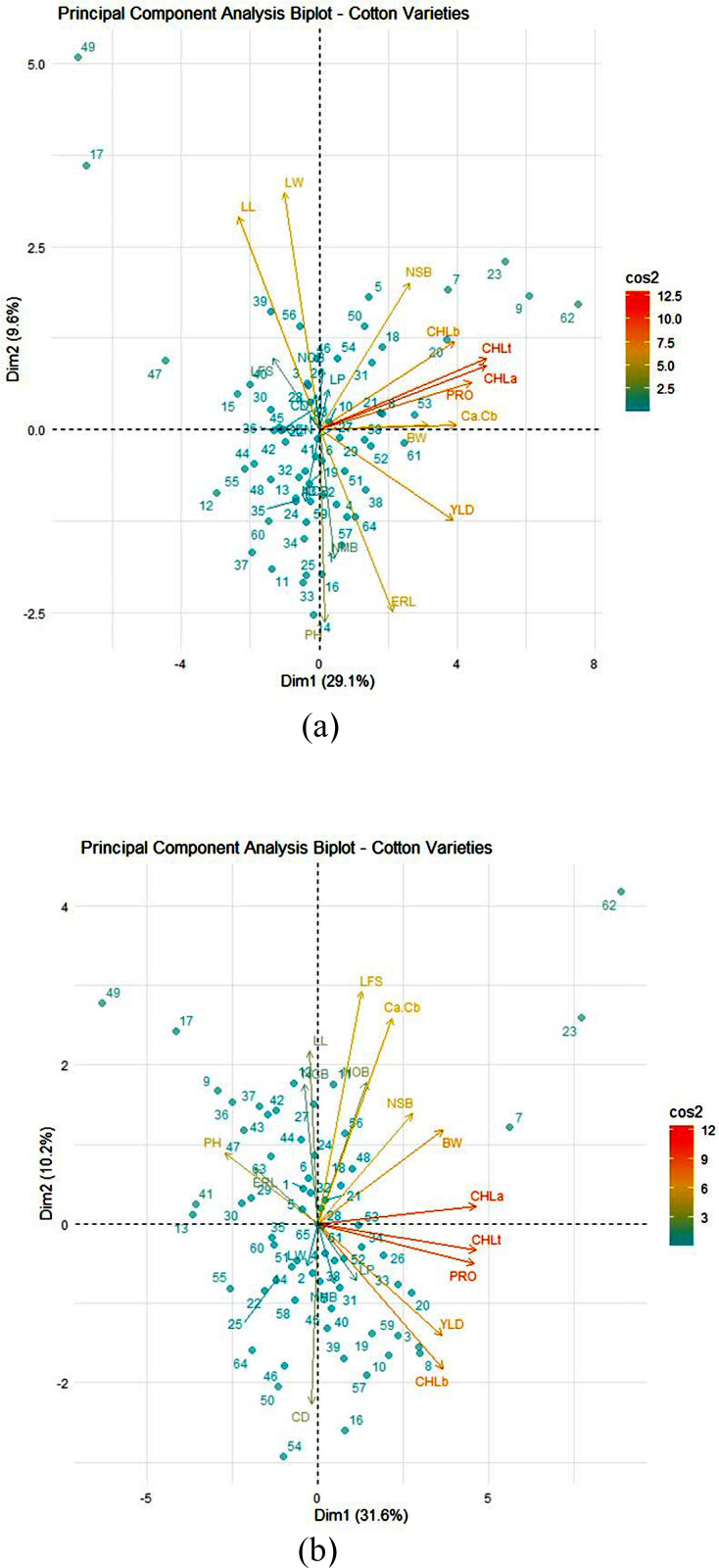
Distribution of the first two principal components (PC) of morphological, agronomic and physiological traits averaged over two years in 64 genotypes of cotton under (a) normal and (b) drought stress conditions in Bardaskan region. BW: boll weight; CD: crown diameter; CHLa: chlorophyll a; CHLb: chlorophyll b; ERL: earliness; LFS: length of fifth sympodial; LL: leaf length; LP: lint percentage; LW: leaf width; NCB: number of closed bolls; NMB: number of monopodial branches; NOB: number of opened bolls; NSB: number of sympodial branches; PH: plant height; PRO: proline content; RAB: ratio of chlorophyll a to chlorophyll b; TCHL: total chlorophyll.

In both regions and moisture regimes, principal component analysis was performed using phenological, agronomic, and physiological traits averaged over two years (Figs 2 and 3). In Kashmar, the first two components explained 47.60% of total variance under normal conditions and 49.50% in drought stress conditions, respectively. Under normal conditions, the first principal component (PC1), which explained 33.40% of the variation, focused on yield, FLP, NMB, CHL a, CHL b, TCHL, and PRO. As a result, this component was classified as a “productivity and photosynthesis ability component”. The second principal component (PC2), which explained 14.20% of the variation, exhibited a positive connection with NCB, LL, PH, and ERL, but a negative correlation with CD, and so might be called the ‘phenological component’. Thus, choosing genotypes with high values in PC1 and PC2 can boost yield and promote early maturity. Genotypes 17, 23, and 49 were identified as high-yielding and drought-tolerant candidates suitable for future breeding programs. (Fig 2a). Under stress conditions, PC1 is heavily weighted for yield, its components, and photosynthetic pigments (CHL a, CHL b, and TCHL), and is known as the “productivity and photosynthesis potential” factor. The PC2 exhibited a positive association with ERL but a negative correlation with yield and its components, which may be interpreted as a difference between productivity and timeliness. Thus, choosing genotypes with high PC1 and low PC2 can increase cotton productivity and earliness. In this respect, genotypes 7, 13, 17, 26, 33, 51, 61, and 64 were the best ones having high production and earliness (Fig 2b).

In Bardaskan, the first two components explained 40.80% of total variation in normal environment and 41.80% under drought stress environment, respectively. Under normal situation, the first principal component (PC1) which explained 30.70% of the variation, had positive connection with the yield, and photosynthesis pigments. As a result, this component was classified as a “productivity and photosynthesis ability component”. The second principal component (PC2) which justified 10.10% of the variance, had a positive association with LL and ERL, and a negative association with ERL, and hence could be called the ‘phenological component’. Thus, choosing genotypes with high values in PC1 and PC2 can boost yield and promote early maturity. In this regard, genotypes 17 and 49 outperformed others due to their high yield and early maturity (Fig 3a). Under stress conditions, PC1 is heavily weighted for yield, its components, and photosynthetic pigments (CHL a, CHL b, and TCHL), and is known as the “productivity and photosynthesis potential” factor. The positive correlation between photosynthetic pigments and yield highlights the physiological basis of drought tolerance. The PC2 demonstrated a positive association with ERL and PH, but a negative correlation with yield and its components, indicating a discrepancy between productivity and earliness. Thus, choosing genotypes with high PC1 and low PC2 can increase cotton productivity and earliness. In this regard, genotypes 23, 30, and 55 outperformed others due to their high production and earliest maturity (Fig 3b).

## 4. Discussion

The availability of genetic variety and the discovery of better characteristics under drought stress conditions for screening drought-tolerant genotypes are critical components of plant development projects. In this study main focus was on the drought tolerance of Iranian germplasm of cotton. These outcomes collectively demonstrate the potential of Iranian cotton germplasm as a valuable genetic resource for drought tolerance. Significant variations were found across genotypes for all evaluated variables in the current study, indicating that there is significant genotypic diversity among cotton genotypes investigated under both normal and drought stress circumstances. This allows for the selection of genotypes with varying drought tolerance. The results indicated significant differences among genotypes and moisture regimes for all traits evaluated. Most of the features changed dramatically over time. This was predicted for phenological parameters, growth measures, and crop load estimations since they are either age-dependent or directly related to heat buildup, which fluctuates yearly [17].

In most agricultural plants, the primary breeding goal is to increase the crop’s production potential [31]. The large genetic diversity among genotypes indicates considerable potential for breeding improvement. Drought stress reduced all characteristics considerably in both locations, with the exception of NCB and PRO in Kashmar and RAB and PRO in Bardaskan. These findings are consistent with those of Singh et al. [4], Abdelraheem et al. [32], and Witt et al. [33] in cotton. A high GCV for a characteristic typically indicates the prospect of improvement through selective breeding. The narrower the gap between PCV and GCV, the lesser the environmental effect. In this study, lower disparities between these two coefficients were found for CHL a, CHL b, TCHL, and PRO, indicating that selecting for these characteristics will result in more benefit. Similar results were reported by Saeidnia et al. [34,35] in orchardgrass and smooth bromegrass.

Heritability assessment, which measures the influence of environmental and genetic variables on the characteristics of interest in order to assess selection efficiency, is required to develop and conduct an effective breeding program to optimize genetic improvement [36,37]. In both locations, most traits displayed high heritability under both conditions, suggesting the presence of key genes or QTLs influencing them [38,39]. These qualities might be enhanced by recurrent or mass selection. For the majority of the measured traits, heritability estimates were higher in normal conditions than in drought stress, which was beneficial for successful selection in achieving genetic progress and suggests that phenotypic selection under normal conditions would be more effective than stress conditions. This is most likely due to alterations in gene expression produced by drought-induced changes in environmental variables that modify plant metabolism and growth [40].

Correlation coefficients are crucial statistical measures for determining the relationship between variables [41], and they are linked to the potential efficacy of indirect selection for complex characteristics [42]. On the other hand, selection might be used on a highly heritable characteristic that is connected to a more complicated trait, such as yield, which has a lower heritability. The results of the phenotypic correlation coefficients, which were confirmed using the PCA approach, demonstrated that yield was positively and substantially linked with PH, BW, NOB, NSB, CHL a, CHL b, and TCHL under both normal and drought stress conditions. Based on the positive and strong correlation between yield and these qualities, as well as their high heritability, it indicates that these traits may be regarded the key components of yield in cotton, and that indirect selection for them could be useful for yield enhancement. On the other hand, the negative association between earliness and yield and its components revealed that earlier blooming plants performed poorly.

The broad distribution of cotton genotypes on the PCA biplot suggested that this germplasm included a high level of genetic diversity in terms of morphological, physiological, and agronomic variables. The results of PCA based on the average of two years indicated that, in both regions of Kashmar and Bardaskan, there were negative associations between yield, its components (PH, CD, NOB, NSB, NMB, and BW), and photosynthetic pigments (CHL a, CHL b, and TCHL), and a positive correlation between yield and photosynthetic pigments under both normal and drought stress conditions, indicating that the earlier flowering genotypes have more photosynthesis ability and are more productive.

In conclusion, significant genetic variation was found in productivity, its components, photosynthetic pigments, and drought tolerance. This suggests that there is a considerable potential for genetic improvement in this material by targeted selection in breeding programs. Water stress has a significant impact on physiological activities, including plant development and biomass output and reduced the genotypic variation of measured traits. The somewhat high heritability estimates for the majority of the variables in this study indicated that repeated selection might possibly be successful. Early-flowering genotypes demonstrated superior productivity, drought tolerance, and stability. This reaction might be attributable to the genotypes’ potential drought avoidance. Based on the researched attributes and the PCA approach, genotypes 17, 23, and 49 were selected as more productive and reasonably tolerant genotypes, which may be employed in future breeding efforts. These contrasting genotypes may serve as valuable parents for developing genetic populations for drought-tolerance research in cotton.

## Supporting information

S1 File Raw data. Raw data for all morphological, agronomic, and physiological traits measured in 64 Iranian cotton genotypes under normal and drought stress conditions in Kashmar and Bardaskan regions.
(XLSX)

S2 File Field photographs and parental genotype information. Photographs taken from experimental cotton fields in Kashmar and Bardaskan regions, along with the names and origins of parental genotypes used in this study.
(DOCX)

## Abbreviations

BW: boll weight
CD: crown diameter
CHLa: chlorophyll a
CHLb: chlorophyll b
ERL: earliness
GCV: genotypic coefficient of variation
LFS: length of fifth sympodial
LL: leaf length
LP: lint percentage
LW: leaf width
NCB: number of closed bolls
NMB: number of monopodial branches
NOB: number of opened bolls
NSB: number of sympodial branches
PCV: phenotypic coefficient of variation
PH: plant height
PRO: proline content
RAB: ratio of chlorophyll a to chlorophyll b
TCHL: total chlorophyll
