# Supplementary material for: Evaluation of diversity and effective traits in Iranian cotton germplasm
Source: PLoS One. 2026 Apr 8;21(4):e0340581. doi: 10.1371/journal.pone.0340581 (PMC13061262; doi:10.1371/journal.pone.0340581)
Supplement: S2 File — Photographs taken from experimental cotton fields in Kashmar and Bardaskan regions, along with the names and origins of parental genotypes used in this study. (DOCX) [file pone.0340581.s002.docx]

| Table 1S- The origin and information of plant genetic materials used in the study | | | |
| --- | --- | --- | --- |
| Male parent | Female parent | origin | Genotype |
| Bakhtegan | Armaghan | Arm×Bak | 1 |
| Hekmat | Armaghan | Arm×Hek | 2 |
| Khordad | Armaghan | Arm×Kho | 3 |
| May | Armaghan | Arm×May | 4 |
| Dr. Omomi | Armaghan | Arm×Omo | 5 |
| T14 | Armaghan | Arm×T14 | 6 |
| Varamin | Armaghan | Arm×Var | 7 |
| Selection on the imported variety called 43347 | | Armaghan | 8 |
| Armaghan | Bakhtegan | Bak×Arm | 9 |
| Hekmat | Bakhtegan | Bak×Hek | 10 |
| Khordad | Bakhtegan | Bak×Kho | 11 |
| May | Bakhtegan | Bak×May | 12 |
| Dr. Omomi | Bakhtegan | Bak×Omo | 13 |
| T14 | Bakhtegan | Bak×T14 | 14 |
| Varamin | Bakhtegan | Bak×Var | 15 |
| Selection on the foreign variety Acala sj2 | | Bakhtegan | 16 |
| Cross between Tadla 6 and Tadla 9 | | Dr.Omomi | 17 |
| Armaghan | Hekmat | Hek×Arm | 18 |
| Bakhtegan | Hekmat | Hek×Bak | 19 |
| Khordad | Hekmat | Hek×Kho | 20 |
| May | Hekmat | Hek×May | 21 |
| Dr. Omomi | Hekmat | Hek×Omo | 22 |
| T14 | Hekmat | Hek×T14 | 23 |
| Varamin | Hekmat | Hek×Var | 24 |
| Selection on imported population called T2 | | Hekmat | 25 |
| Armaghan | Khordad | Kho×Arm | 26 |
| Bakhtegan | Khordad | Kho×Bak | 27 |
| Hekmat | Khordad | Kho×Hek | 28 |
| May | Khordad | Kho×May | 29 |
| Dr. Omomi | Khordad | Kho×Omo | 30 |
| T14 | Khordad | Kho×T14 | 31 |
| Varamin | Khordad | Kho×Var | 32 |
| Selection on an imported population called Sinduz | | Khordad | 33 |
| imported variety from Turkey | | May | 34 |
| Armaghan | May | May×Arm | 35 |
| Bakhtegan | May | May×Bak | 36 |
| Hekmat | May | May×Hek | 37 |
| Khordad | May | May×Kho | 38 |
| Dr. Omomi | May | May×Omo | 39 |
| T14 | May | May×T14 | 40 |
| Varamin | May | May×Var | 41 |
| Armaghan | Dr. Omomi | Omo×Arm | 42 |
| Bakhtegan | Dr. Omomi | Omo×Bak | 43 |
| Hekmat | Dr. Omomi | Omo×Hek | 44 |
| Khordad | Dr. Omomi | Omo×Kho | 45 |
| May | Dr. Omomi | Omo×May | 46 |
| T14 | Dr. Omomi | Omo×T14 | 47 |
| Varamin | Dr. Omomi | Omo×Var | 48 |
| imported variety from Egypt | | T14 | 49 |
| Armaghan | T14 | T14×Arm | 50 |
| Bakhtegan | T14 | T14×Bak | 51 |
| Hekmat | T14 | T14×Hek | 52 |
| Khordad | T14 | T14×Kho | 53 |
| May | T14 | T14×May | 54 |
| Dr. Omomi | T14 | T14×Omo | 55 |
| Varamin | T14 | T14×Var | 56 |
| Armaghan | Varamin | Var×Arm | 57 |
| Bakhtegan | Varamin | Var×Bak | 58 |
| Hekmat | Varamin | Var×Hek | 59 |
| Khordad | Varamin | Var×Kho | 60 |
| May | Varamin | Var×May | 61 |
| Dr. Omomi | Varamin | Var×Omo | 62 |
| T14 | Varamin | Var×T14 | 63 |
| Cross between Coker 100 Wilt and Strain 539 | | Varamin | 64 |


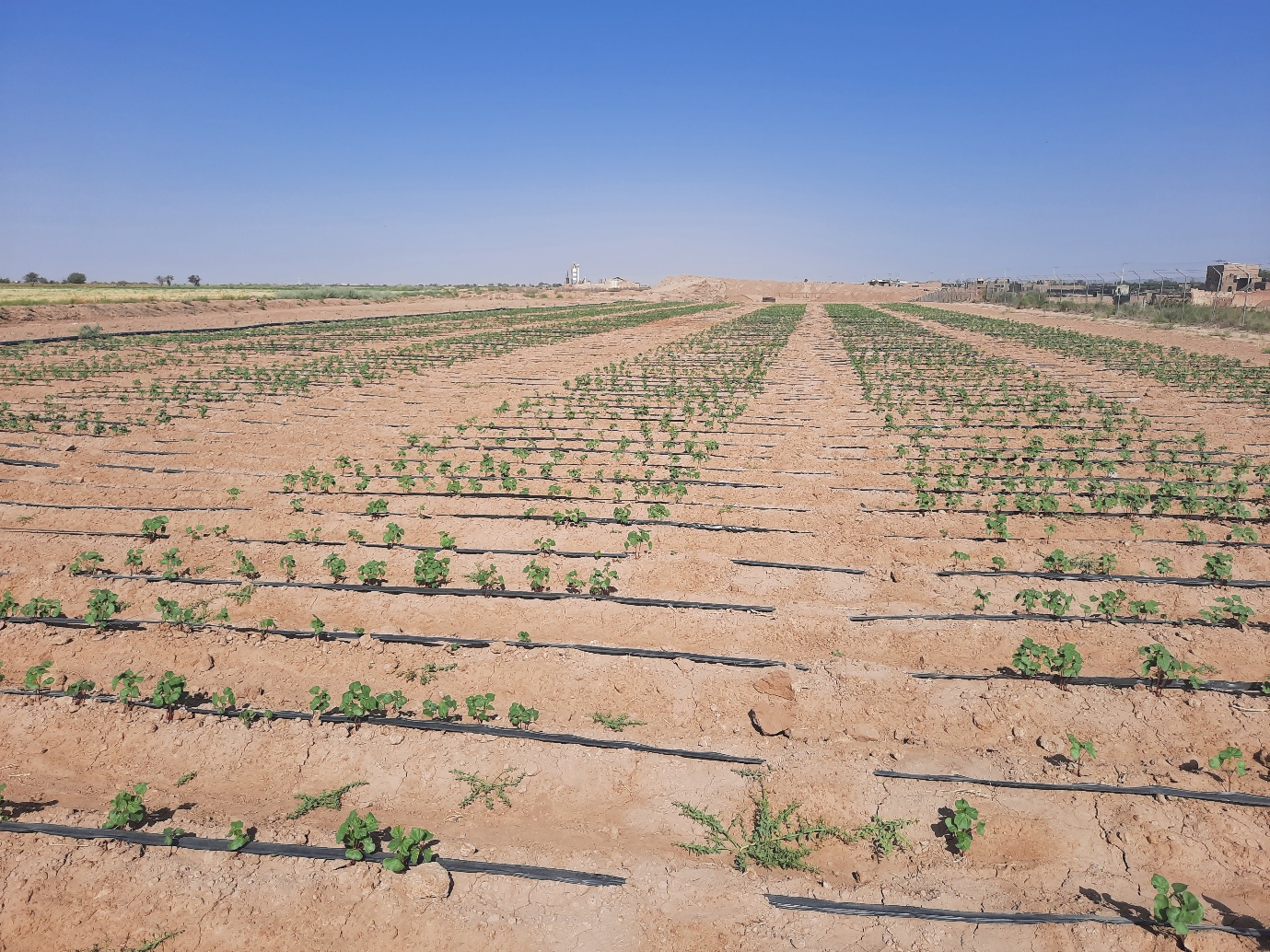


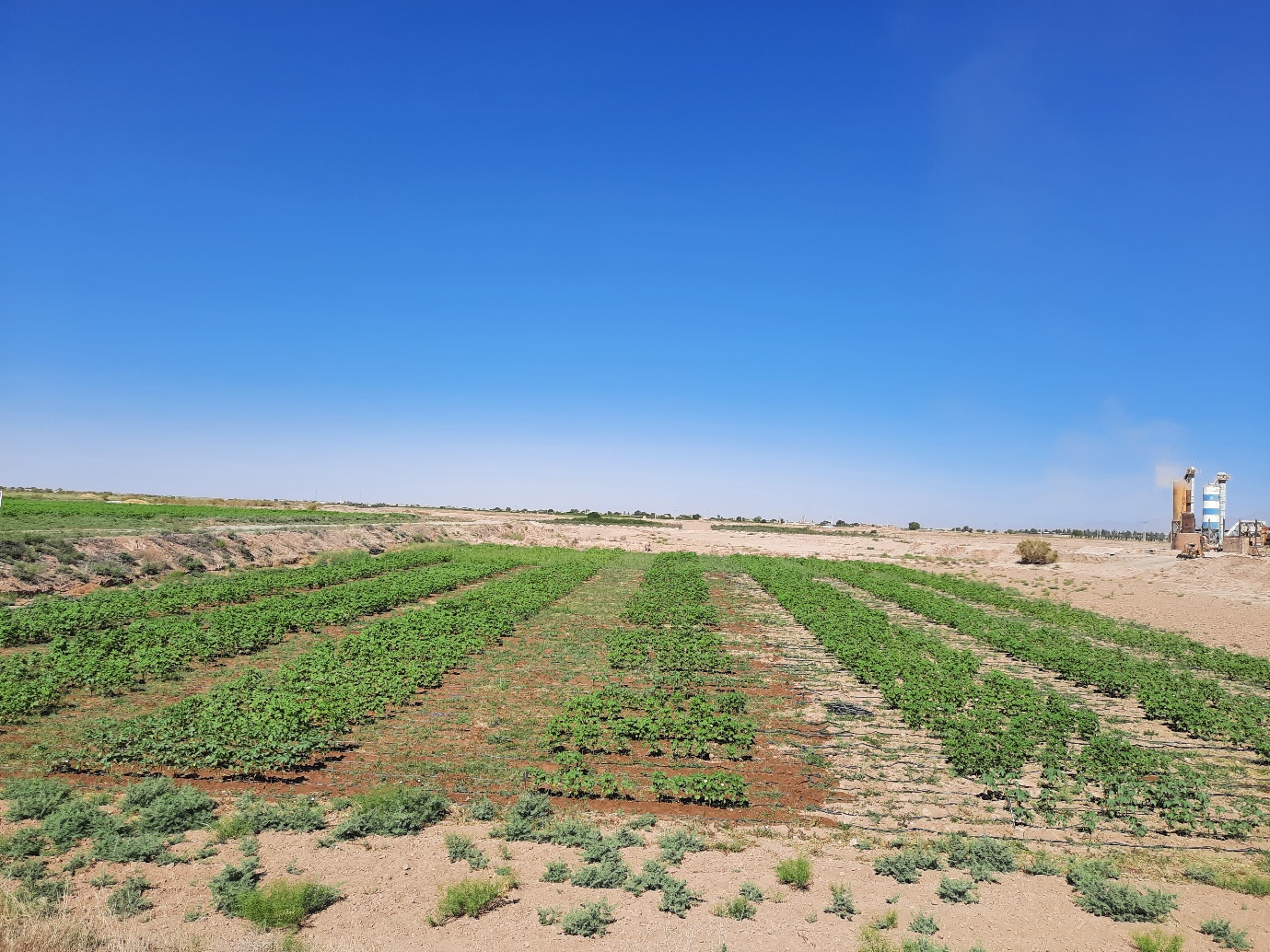


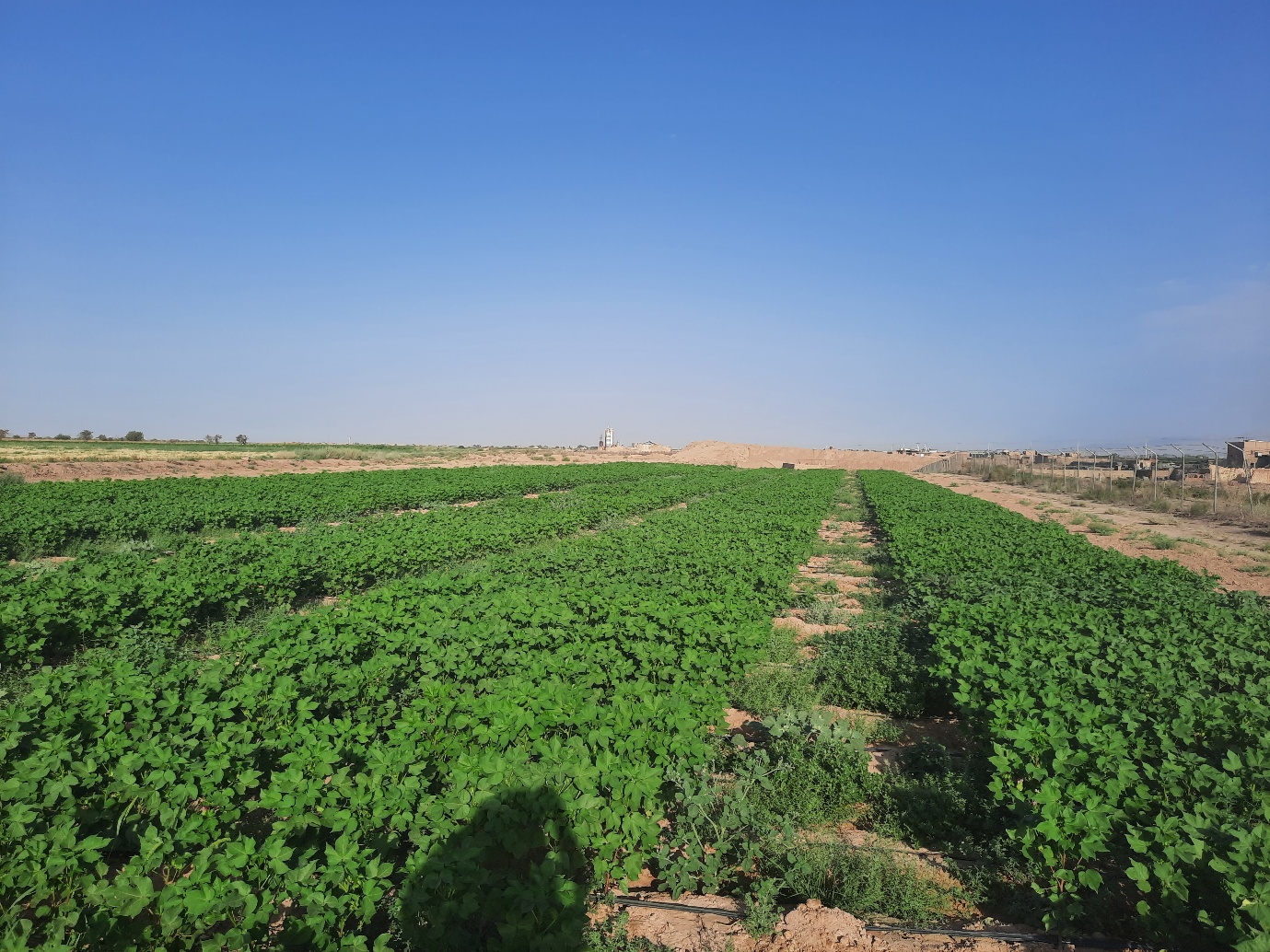


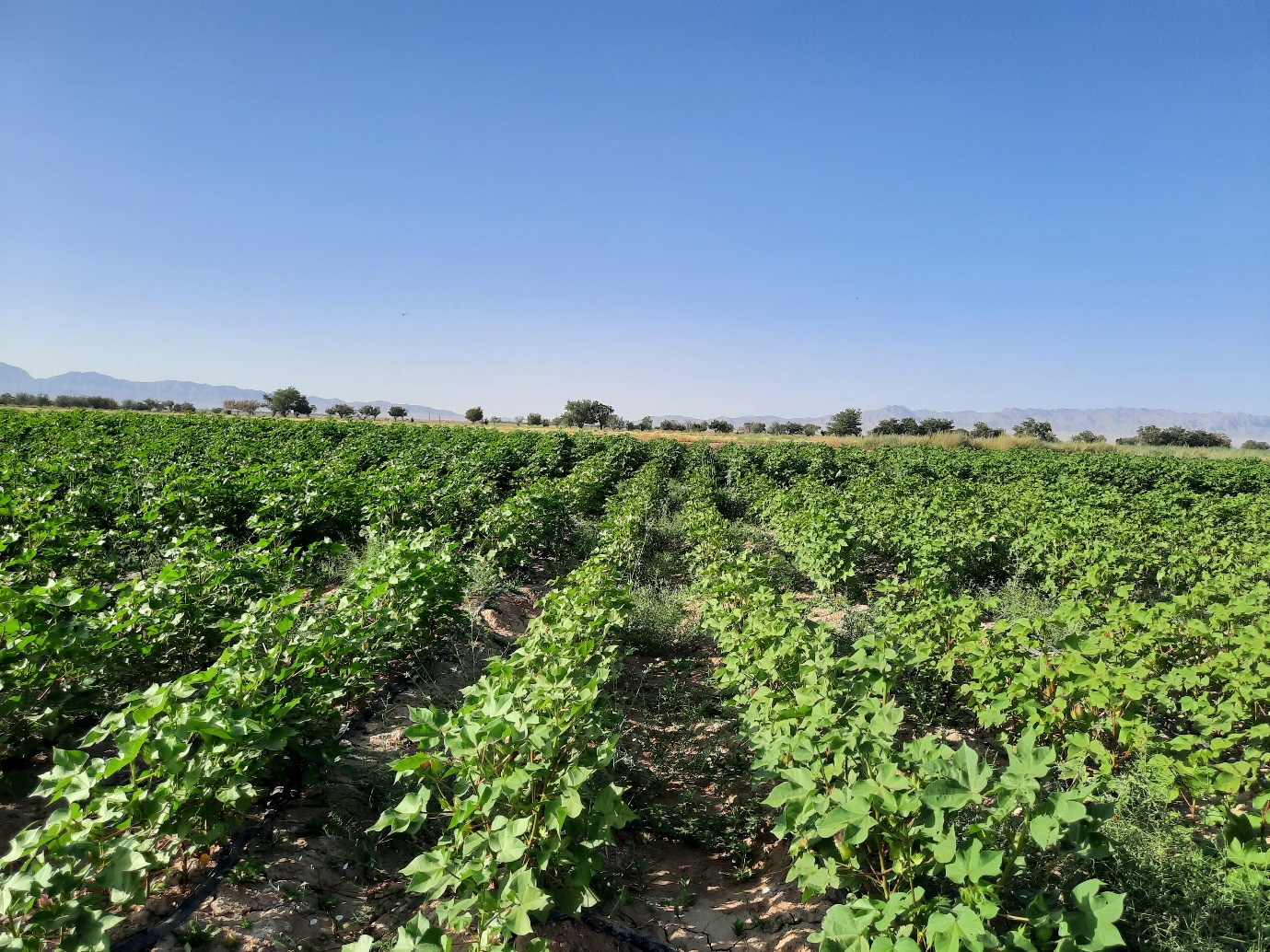


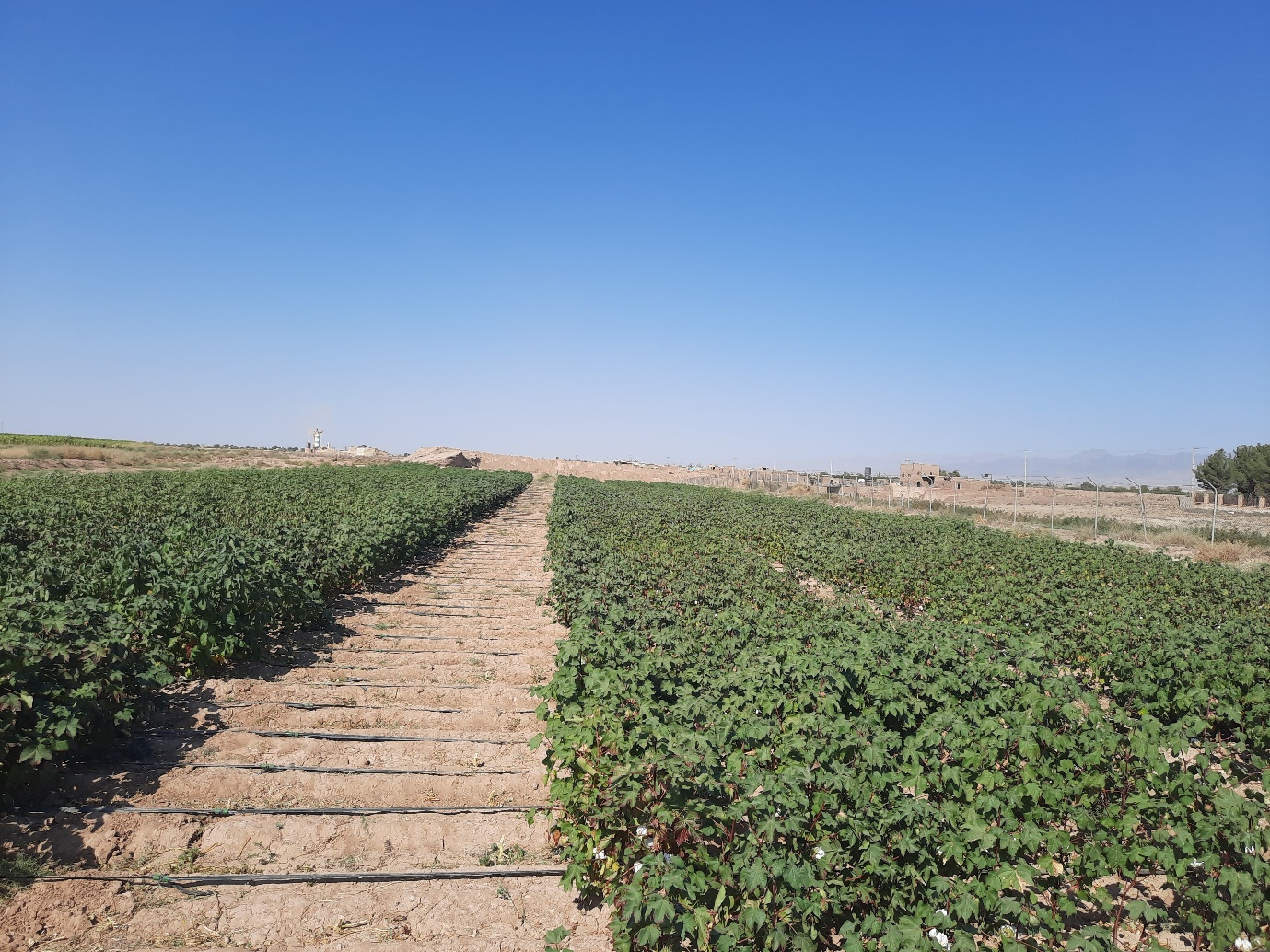


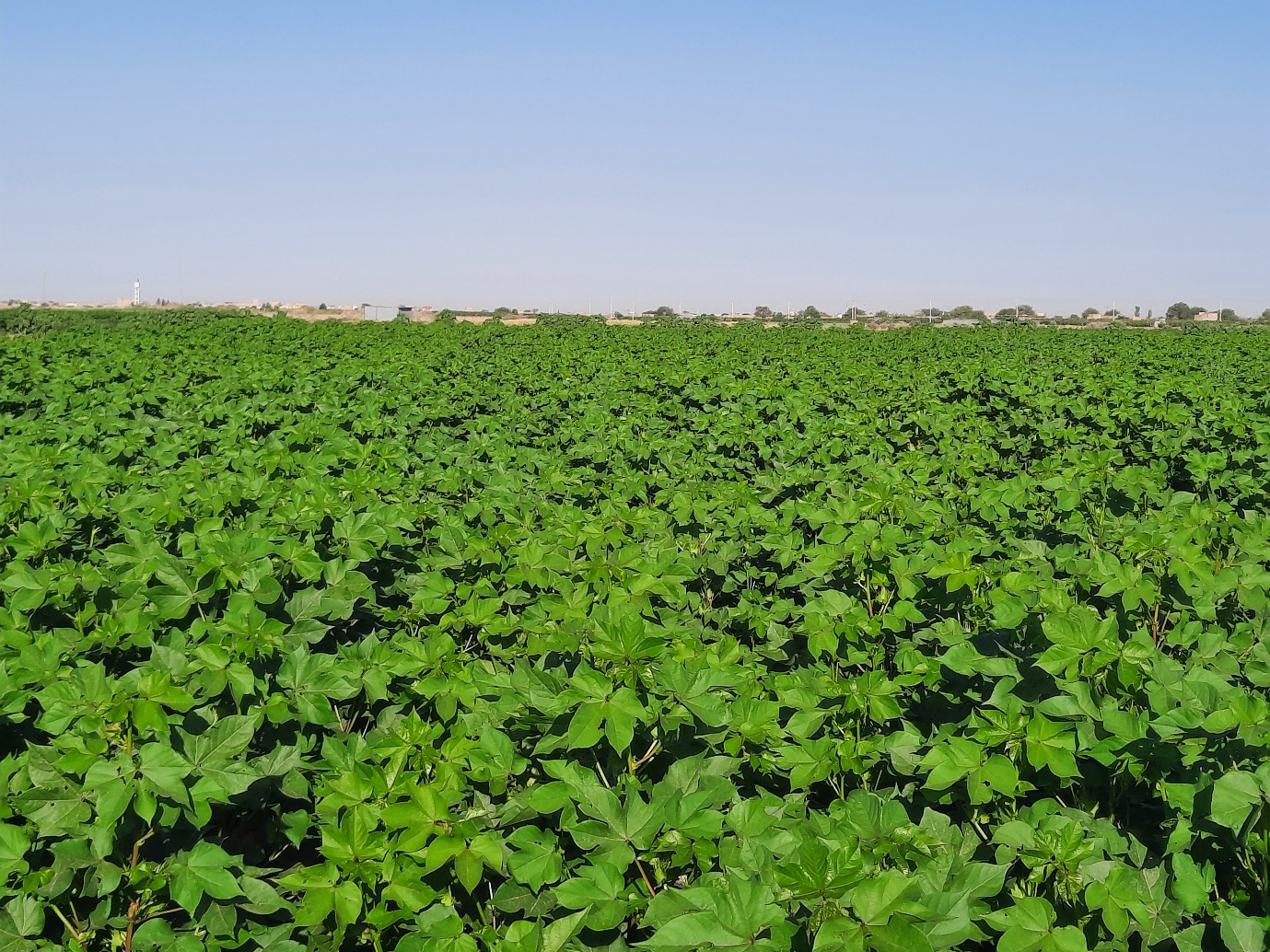


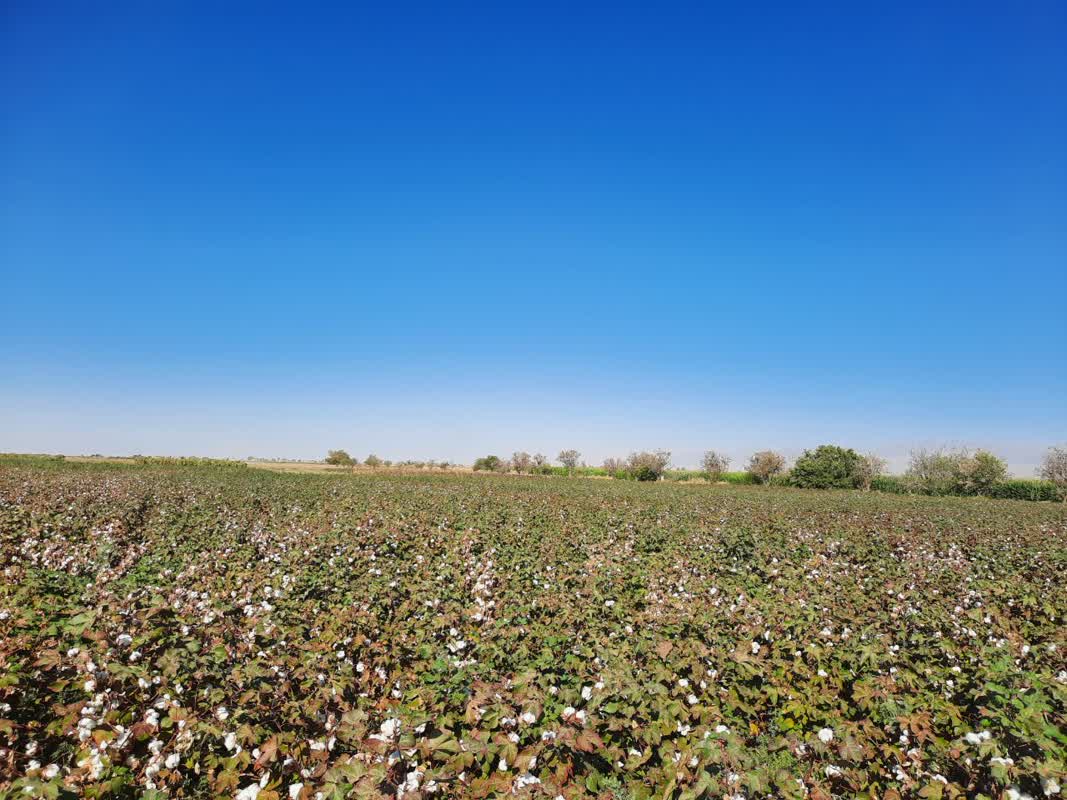


**Fig S1-** Schematic view of the field experiment from planting stage to harvesting.
